# Antibiotics and antifungals in silicone oil

**DOI:** 10.1186/s40942-019-0199-2

**Published:** 2019-12-12

**Authors:** Ella H. Leung, J. Timothy Stout

**Affiliations:** Cullen Eye Institute, Baylor College of Medicine, 1977 Butler Blvd, Houston, TX 77030 USA

**Keywords:** Silicone oil, Antibiotics, Antifungals

## Abstract

**Background:**

Antimicrobials may be injected into silicone oil-filled eyes with endophthalmitis, but the interaction with oil is unclear. The purpose of the experiment is to determine whether vancomycin, amikacin, and amphotericin B mix with silicone oil.

**Methods:**

Using the relative proportions of the human eye, 4 ml of 1000 centistokes silicone oil was centrifuged with 0.1 ml of vancomycin 1 mg/0.1 ml, amikacin 0.4 mg/0.1 ml, or amphotericin B 5 µg/0.1 ml in vitro and eluted. The aqueous was immediately analyzed with a liquid chromatographer/mass spectrometer and after 24 h.

**Results:**

Within 24 h, a mean of 26.9 μmol/L of vancomycin, 0 nmol/L of amikacin, and 0 nmol/L of amphotericin B were recovered. When the concentrations of amikacin and amphotericin B were increased 100-fold, 0 nmol/L of amikacin and 75.7 µmol/L of amphotericin B were recovered.

**Conclusions:**

Vancomycin and amphotericin B partially mixed with the silicone oil. Amikacin was not recovered from the antibiotic–silicone oil mixture.

## Background

Vancomycin, amikacin, and amphotericin B are among the most commonly used intravitreal antibiotics and antifungal agents for the treatment of endophthalmitis. Although intravitreal injections are effective, patients with severe infections or concurrent retinal detachments may benefit from pars plana vitrectomy (PPVs) with silicone oil (SO) tamponades to decrease the bacterial or fungal load, remove inflammatory mediators, and reattach the retina [1]. Previously published studies have reported better visual and anatomic outcomes with PPVs and silicone oil compared to core vitrectomies alone in patients with endophthalmitis.

Silicone oil has been reported to decrease bacterial and fungal survival in vitro. The oil was effective in decreasing the microbial loads of *Staphylococcus aureus, Staphylococcus epidermidis, Pseudomonas aeruginosa*, *Escherichia coli, Fusobacterium* spp., *Clostridium tertium, Peptotstreptococcus* spp., *Bacteroides fragilis*, *Candida albicans,* and *Aspergillus* spp. [2–5]. It is theorized that the viscous oil acts as a physical barrier to deprive the bacteria and fungus of nutrition, and the catalysts and components of the oil may be toxic to the microbes; however, the growth of certain anaerobic bacteria like *Propionibacterium acnes* and yeast like *Candida albicans* may not be fully inhibited by the oil [2, 3].

The safe and efficacious dose of intravitreal antimicrobials in eyes filled with silicone oil depends on multiple factors, including the dose administered and the amount that may be secluded within the oil or compartmentalized against the retina [6–8]. Retinal toxicity has been reported with ceftazidime 2.25 mg/0.2 ml and vancomycin 1.25 mg/0.2 ml injected into the 1.5 ml vitreous cavities of rabbit eyes; however, the one-quarter dose was considered safe [7]. Since rabbit eyes are smaller than human eyes, however, a quarter-dose in rabbits is approximately equal to half the usual dose in humans. Many surgeons therefore inject one-half to one-forth of the standard dose of antimicrobials in eyes filled with silicone oil. It is currently unknown whether intravitreal antibiotics and antifungals may mix with the silicone oil itself, thereby becoming a medication reservoir and becoming pharmacologically unavailable.

The purpose of the study is to determine whether vancomycin, amikacin, and amphotericin B mix with silicone oil in vitro.

## Methods

**Fig. 1:**
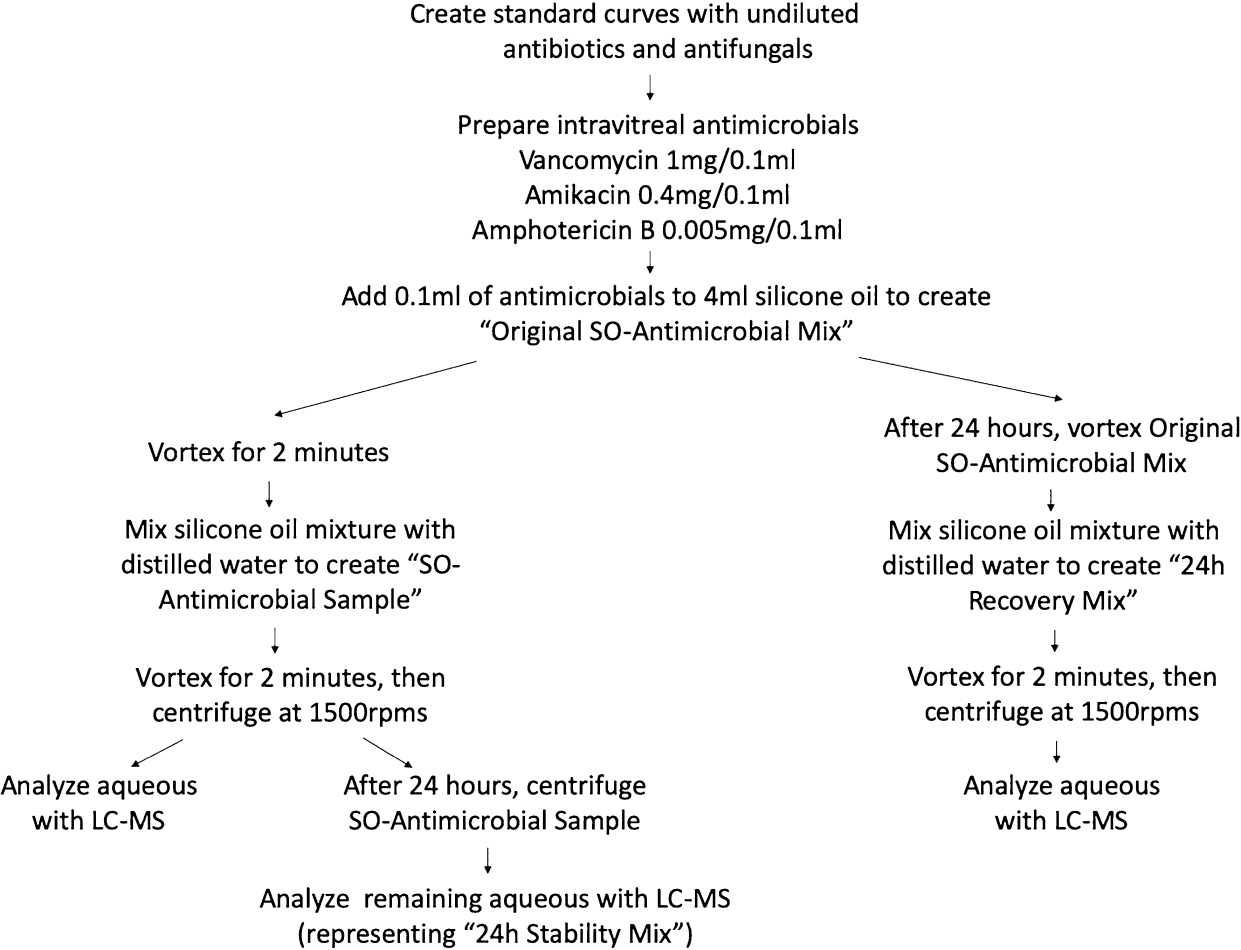
Procedure protocol. Graphical representation of the protocol. The “SO-Antimicrobial Sample” represents the amount of antimicrobial recovered immediately after injecting the antimicrobials into the silicone oil. The “24 h Stability Mix” represents the additional antimicrobials recovered from the “SO-Antimicrobial Sample” after 24 h. The “24 h Recovery Mix” represents the quantity of antimicrobials that had remained in the original silicone oil–antimicrobial mixture and had not diffused out after the first sample

The methodology is outlined in Fig. 1. The study did not involve human subjects so an Institutional Review Board approval was not required. Standard concentration curves were created using pure samples of vancomycin (Mylan Pharmaceuticals, IL), amikacin (Geneva Pharmaceuticals, NJ), and amphotericin B (X-Gen Pharmaceuticals, NY) in a liquid chromatographer—mass spectrometer (Agilent 6490 Triple-Quadropole LC–MS, CA); the correlation coefficients were 0.97–0.99 for all 4 antimicrobials. The LC–MS detected nanomolar concentrations. Pure samples of 0.9% sodium chloride solution (NaCl), 5% dextrose in water (D5W), and distilled water served as controls.

The intravitreal formulations of each medication were compounded. Vancomycin was dissolved in 0.9% NaCl to achieve a concentration of 1 mg/0.1 ml, amikacin in 0.9% NaCl (0.4 mg/0.1 ml), and amphotericin B in D5W (5 μg/0.1 ml).

Using the relative proportions of the vitreous cavity, 4 ml of 1000 centistokes silicone oil (Alcon, Fort Worth, TX) was mixed with 0.1 ml of each of the 3 antimicrobials. The experiment was performed in triplicate, and the results were averaged. The “Original SO-Antimicrobial Mix” was vortexed for 2 min at 15,000 rotations per minute (rpm), and 1.6 ml of only the silicone oil layer was removed, with care taken to not disturb the injected aqueous bubble. Since silicone oil cannot be directly analyzed with the LC–MS, the antibiotics were eluted out of the oil mixture by centrifuging with 1.6 ml of distilled water for 2 min at 15,000 rpms. Distilled water was used as a solvent because balanced salt solution can cause artifacts and other organic solvents can bind with the silicone oil molecules. The aqueous layer from this “SO-Antimicrobial Sample” was immediately analyzed with the LC–MS.

After 24 h, the remainder of the “SO-Antimicrobial Sample” was vortexed for 2 min, and the aqueous analyzed with the LC–MS; the new sample represented the stability of antimicrobials being recovered and was referred to as the “24 h Stability Mix.” The “Original SO-Antimicrobial Mixture” was also vortexed again after 24 h; 1.6 ml of the silicone oil mixture was removed and centrifuged with 1.6 ml of distilled water to elute out remaining antimicrobials; that mixture represented additional recovery of antimicrobials and was referred to as the “24 h Recovery Mix.”

## Results

**Fig. 2:**
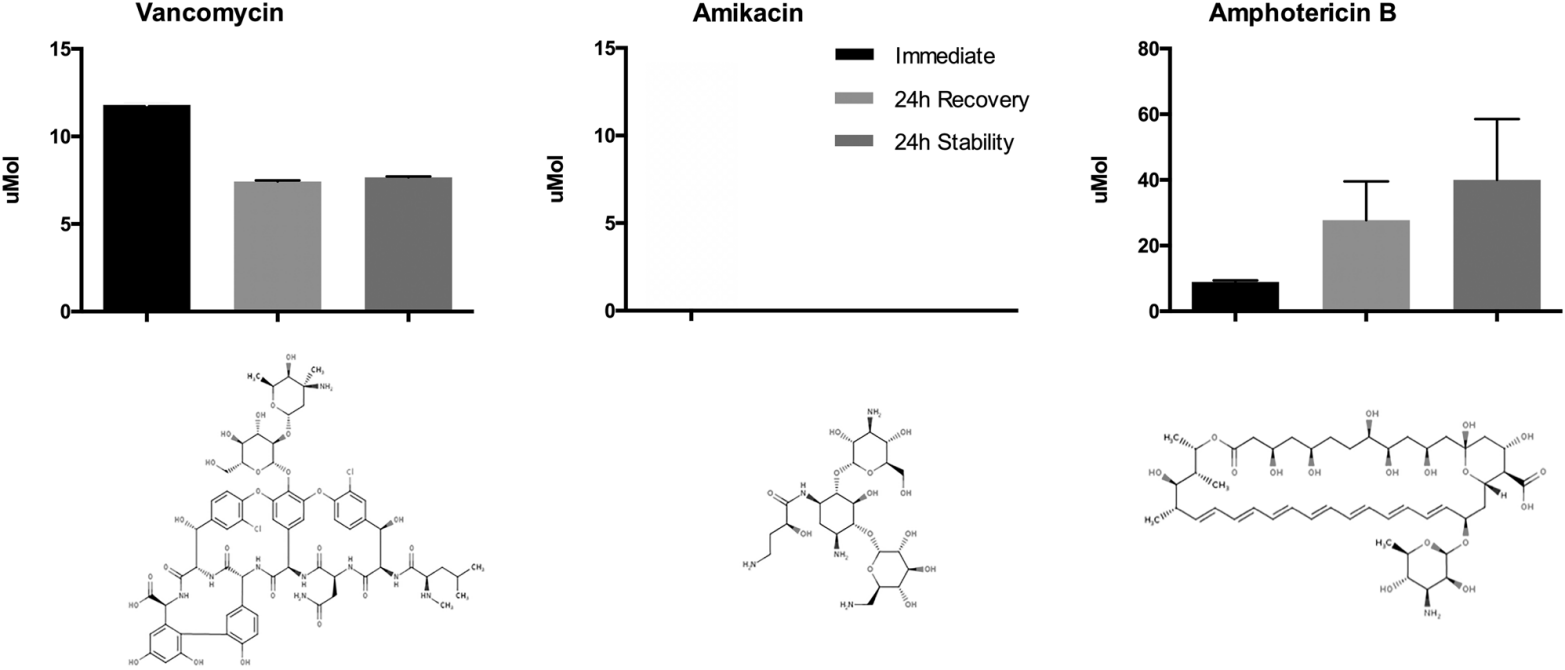
Antibiotic and antifungals recovered from silicone oil. The graph demonstrates that small quantities of vancomycin and amphotericin B mixed with silicone oil. Although none of the amphotericin B was recovered when the intravitreal concentrations were used (5 mcg/0.1 ml), amphotericin B was recovered when 500 mcg/0.1 ml was used

Immediately after mixing the antimicrobials with silicone oil, a mean of 11.8 ± 0.11 μmol/l (µM) of vancomycin, 0 nmol/l (nM) of amikacin, and 0 nM amphotericin B were recovered (Fig. 2). After 24 h, an additional 7.66 ± 0.06 µM vancomycin, 0 nM amikacin, and 0 nM amphotericin B were recovered from the eluted sample, representing the “24 h Stability Mix.” An additional 7.43 ± 0.058 µM vancomycin, 0 nM amikacin, and 0 nM amphotericin B were recovered after 24 h (“24 h Recovery Mix”).

Due to the low concentrations of amikacin and amphotericin B being tested and the heterogeneous properties of the silicone oil-antimicrobial mixture, the experiment was repeated again using 100 times the concentration of amikacin (40 mg/0.1 ml, instead of 0.4 mg/0.1 ml) and amphotericin B (0.5 mg/0.1 ml, instead of 0.005 mg/0.1 ml). While no amikacin was recovered, a mean of 8.62 ± 0.89 µM of amphotericin B was recovered initially from the higher concentration, an additional 40.0 ± 19 µM was obtained from the 24 h stability sample, and 27.7 ± 12 mM from the 24 h recovery sample. Samples where the aqueous bubble had broken up into smaller bubbles had a greater recovery of antimicrobials.

## Discussion

Endophthalmitis is a potentially blinding condition that can occur after intraocular surgery, trauma, or systemic infection. In the Endophthalmitis Vitrectomy Study, only 14% of patients recovered visual acuity better than 5/200 [8]. Therapies to treat endophthalmitis include intravitreal antibiotics, intravitreal antifungals, and pars plana vitrectomies. In a patient with endophthalmitis and concurrent retinal detachment, silicone oil’s potential antimicrobial properties makes it appealing as an intraocular tamponade. Retinal detachments may occur in 4.6–16% of eyes with endophthalmitis after vitrectomies, compared to less than 5% in eyes undergoing vitrectomies for other indications [9]; however, the risk of a retinal detachment may be decreased in eyes with concurrent silicone oil implantation [1, 10].

The in vitro experiments demonstrated that amphotericin B and vancomycin partially mixed with the silicone oil while amikacin did not. The chemical properties may help explain the relative antimicrobial recoveries from silicone oil. Silicone oil is composed of a mixture of organic and inorganic, hydrophobic and inert polymers and siloxanes [4]. Vancomycin is primarily hydrophilic but has hydrophobic elements and rings. Amikacin is primarily hydrophilic. Amphotericin B has a hydrophobic polyene chain and a hydrophilic tail and polar head and may be mixed with other compounds like sodium phosphate to increase water solubility (Fig. 2). The more hydrophobic antimicrobials, like amphotericin B interacted to a greater extent with the oil than the hydrophilic antibiotics, like amikacin.

Due to the in vitro nature of the experiments, it was not possible to apply the results to in vivo clinical scenarios; however, the current experiment did provide information regarding the potential of these antimicrobials to mix with silicone oil. While most of the antimicrobials would have been pharmacologically available, a small amount may mix with silicone oil. Given that intravitreal concentrations of antibiotics and antifungals are typically several orders of magnitude higher than the 90% minimum inhibitory concentrations (MIC90) for most bacterial and fungal organisms (0.016–32 μg/ml), the amount of antimicrobials that would have mixed with the silicone oil would have likely been clinically insignificant [12–14]. The clinician may therefore consider injecting a lower dosage of amikacin since the majority of the injected water-soluble amikacin would be concentrated in the remaining aqueous volume, and amphotericin B may need to be reinjected if the endophthalmitis does not resolve since voriconazole is not fungicidal and may mix with the oil.

The limitations of the current study include the indirect methodology used to quantify the antimicrobials that had partitioned into silicone oil and the low concentrations of antimicrobials used. Some antimicrobials may have been lost in the transfers, diffused out later, were below the limits of detection of the LC–MS, may have diffused out later, or were not mixed homogenously. Silicone oil cannot be directly measured with a LC–MS, so the antimicrobials were eluted out and the concentrations measured on repeat testing. Future studies could include determining the rate at which the antimicrobials are released from silicone oil in vivo, testing the effects of ceftazidime and voriconazole in oil, and using a different type of silicone oil. Unfortunately, ceftazidime was not available during the study due to a manufacturer shortage. The 1000 centistokes silicone oil was used in the current experiments because it is more commonly available, but the more viscous 5000 centistokes silicone oil or heavy silicone oil may be more effective for the complex retinal detachments that may occur with endophthalmitis and in inhibiting bacterial growth [3].

In conclusion, the in vitro experiments demonstrated that a small proportion of vancomycin and amphotericin mixed with silicone oil. Amikacin was not recovered from silicone oil, even after increasing its concentration. It is likely that the remaining unbound antimicrobials would be pharmacologically available.

## Data Availability

The data generated or analyzed in this study are included in this published article and are available from the corresponding author on reasonable request.
